# On the Use of Solution of Caustic Potash in Relieving Stranguary

**Published:** 1848

**Authors:** R. Mulock


### ARTICLE XII.

### *On the Use of Solution of Caustic Potash in relieving Stranguary.*
By R. Mulock, M. D.

In three cases of stranguary, caused by blistering with cantharides, I found the solution of caustic potash a perfect remedy. Two of the cases were head affections, where opium was inadmissible. Thirty drops, given in half a wine-glass of water every hour, gave relief before the third dose was exhibited. What led me to the use of this preparation was its known effects in relieving irritation of the bladder in other cases, and also its efficacy in relieving the stings of wasps or bees when applied to the skin. I considered that it might also relieve the acrid principle of the cantharides. Indeed, from its effects, it would probably be a remedy for an overdose of that medicine, if given in proper time. In looking over the various works on irritative poisons, I find no antidote to the poison of cantharides, and mention this now that further trial may be made of its effects in giving relief.—*Dub. Jour. Med. Sci.*

